# Rare Aggressive Calvarial Osteoblastoma with Dural Invasion

**DOI:** 10.7759/cureus.1733

**Published:** 2017-09-30

**Authors:** Line Jacques, Isabelle Mousseau, Qasim Al Hinai, Junia Santos, Ayoub Nahal, Judith Marcoux

**Affiliations:** 1 Neurosurgery, UCSF; 2 Montreal Neurological Institute, McGill University Health Centre; 3 Pathology and Laboratory Medicine Institute, Cleveland Clinic Abu Dhabi

**Keywords:** aggressive osteoblastoma, local recurrence, en-block resection, calvarial tumors

## Abstract

We report a rare case of an aggressive osteoblastoma (OB) involving the calvaria and infiltrating the dura, a finding that was not previously reported in the literature. A 50-year-old man presented with a progressive mass in the left frontoparietal skull with headaches and a six-month history of sudden mass growth. Computed tomography (CT) and magnetic resonance imaging (MRI) showed a large skull lesion with areas of hemorrhage, calcification, restricted diffusion, and enhancement.  A left temporoparietal craniotomy with a complete resection of the tumor with grossly clean margins was performed. Follow-up at 60 months showed a stable clinical picture and no sign of local recurrence of the lesion on MRI.

## Introduction

Osteoblastoma is a rare benign bone-forming tumor that accounts for less than one percent of all primary bone neoplasms [1] and occurs mostly in childhood and adolescence, more often in males [2]. Although it has a predilection for the axial skeleton, osteoblastoma is rarely seen in the calvaria [3]. There have been 47 cases of osteoblatoma in the cranial vault, one of the most commonly reported locations for this kind of tumor in the skull [4], reported in the English literature [1, 5-10].

Based on our literature research, only eight cases were related to calvarial aggressive osteoblastomas, when considering the frontal, parietal, temporal, and occipital bones as true calvarium [6]. We report a rare case of an aggressive osteoblastoma involving the calvaria and infiltrating the dura, a finding that was not previously reported in the literature.

## Case presentation

A 50-year-old man presented with a progressive mass in the left frontoparietal skull with headaches and a six-month history of sudden mass growth of a previously stable left subcutaneous parietal mass present since a fall at the age of six years. Computed tomography (CT) and magnetic resonance imaging (MRI) of the brain showed a large skull lesion with areas of hemorrhage, calcification, restricted diffusion, and enhancement. Intraoperative findings showed dural infiltration through the cortical side and large vessels from the cortex feeding the infiltrated dura. The histological examination revealed an aggressive osteoblastoma (OB) with a secondary aneurysmal bone cyst.

His past medical history included coronary artery disease stented the year prior, hypertension, and remote bowel surgery for a perforated viscus. Physical examination revealed a firm, non-tender, slightly mobile mass in the left parietal area. Cranial nerves were intact, there was no pronator drift, and sensation and power were normal in all limbs.

**Figure 1:**
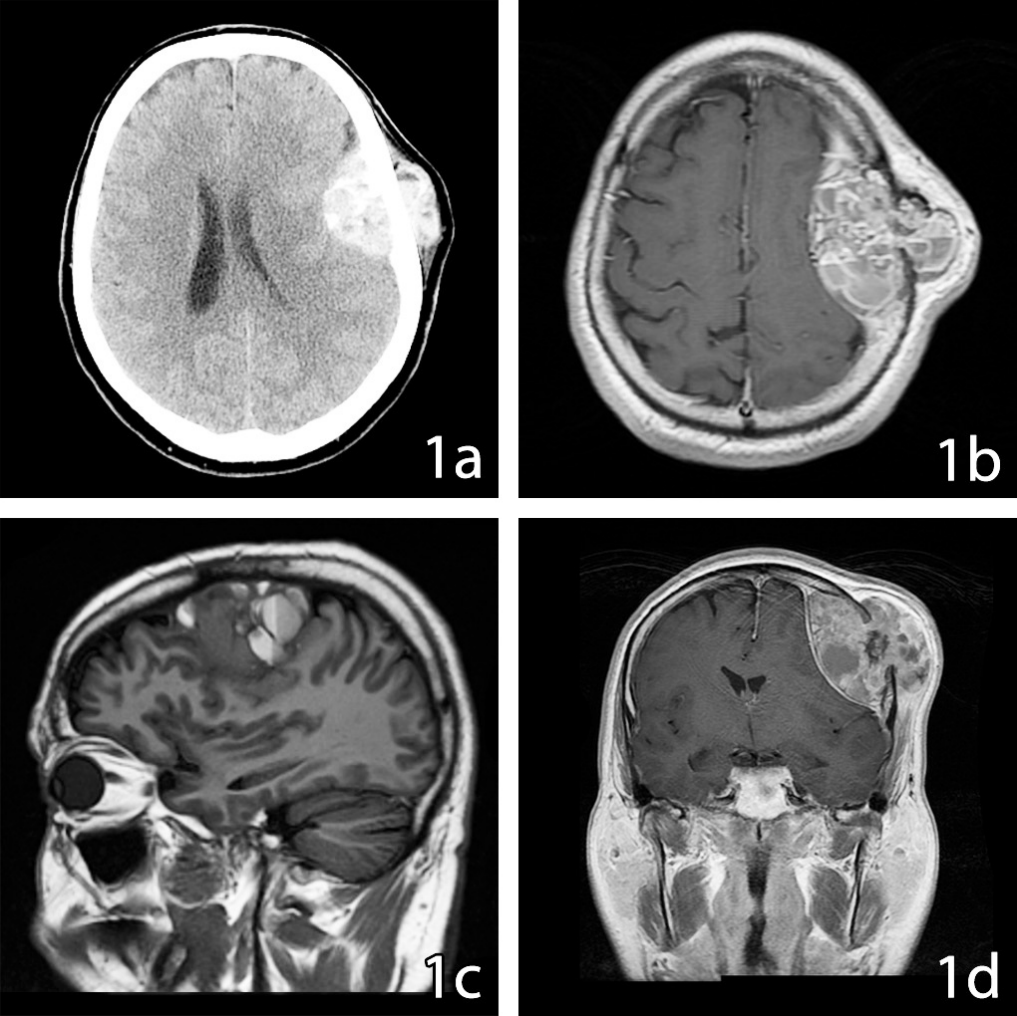
Skull Tumor Aggressive osteoblastoma imaging before surgical resection. 1a: Axial CT. 1b: Axial T1-weighted MR. 1c: Parasagital T1-weighted MR. 1d: Coronal T1-weighted MR. High attenuation mass with intracranial and extracranial components on CT (1a) appears well circumscribed. The lesion has heterogeneous intensity on T1-weighted MR (1b), predominantly hyperintense with isointense areas, compromises the integrity of left parietal bone (1c) and produces a slight midline shift to the right (1d). CT: computed tomography MR: magnetic resonance

Preoperative radiological evaluation included CT with and without contrast, and MRI (Figure 1).

**Figure 2:**
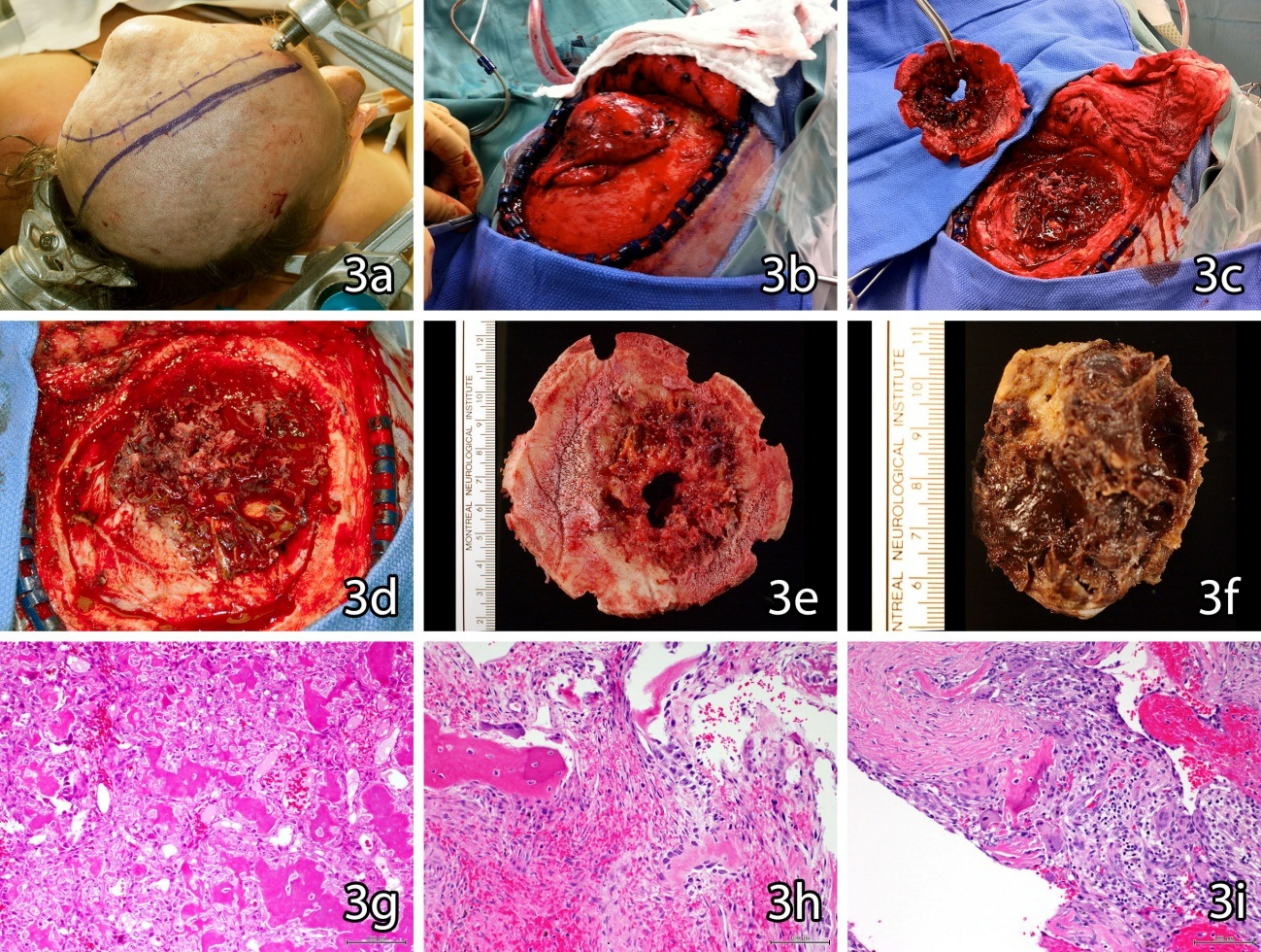
Histology Histopathological findings from aggressive osteoblastoma. 3a: External aspect of the left parietotemporal tumor. 3b: Intraoperative gross aspect. 3c: Intraoperative aspect after bone resection. 3d: Intraoperative aspect of underlying dura mater. 3e: Bone flap left fronto-temporo-parietal specimen for gross pathology analysis. 3f: Left fronto-temporo-parietal tumor with subjacent dura mater. 3g/h:  Hematoxylin and eosin (H&E) staining of tumor sections. 3i: H&E staining of underlying dura mater. Macroscopically, the lesion presented large hemorrhagic areas, calcified nodules and bony spicules, including a yellowish connective tissue on the outer aspect, and adipose-like grey tissue. The dura appeared attached to both the intracranial portion of the mass and to the underlying cortex through numerous large vessels. Intralesional cystic change was present and corresponds to secondary aneurismal cyst. The intraoperative evaluation suggested an osteosarcoma, according to its aggressiveness and osteolytic and infiltrative features. However, the histological evaluation denoted a highly cellular lesion, composed by sheets of epithelioid osteoblasts with rare mitotic figures (3g/h/i) and absence of sarcomatous proliferation, establishing the diagnosis of aggressive osteoblastoma.

The solitary destructive left frontoparietal bone lesion was made of intracranial (3.9 x 6.7 x 4.6 cm) and extracranial components (4.7 x 2.4 cm) destroying both the inner and outer tables. The dura directly under the mass showed a signal change and thinning. The mass was heterogeneous with loculations and septations, fluid-line, and hemorrhagic or calcified areas, with no evidence of surrounding brain edema. There was a 7 mm midline shift. A chest CT showed several lung nodules measuring up to 8 mm that were eventually deemed not related to the cranial lesion. The metastatic workup, as well as blood tumor markers, were negative. The vascularization of the tumor was assessed through an angiogram that revealed a well-vascularized lesion with shared blood supply between the middle meningeal artery and the superficial temporal artery. Branches of the middle meningeal artery were embolized the day prior to surgery (Figure 2).

**Figure 3:**
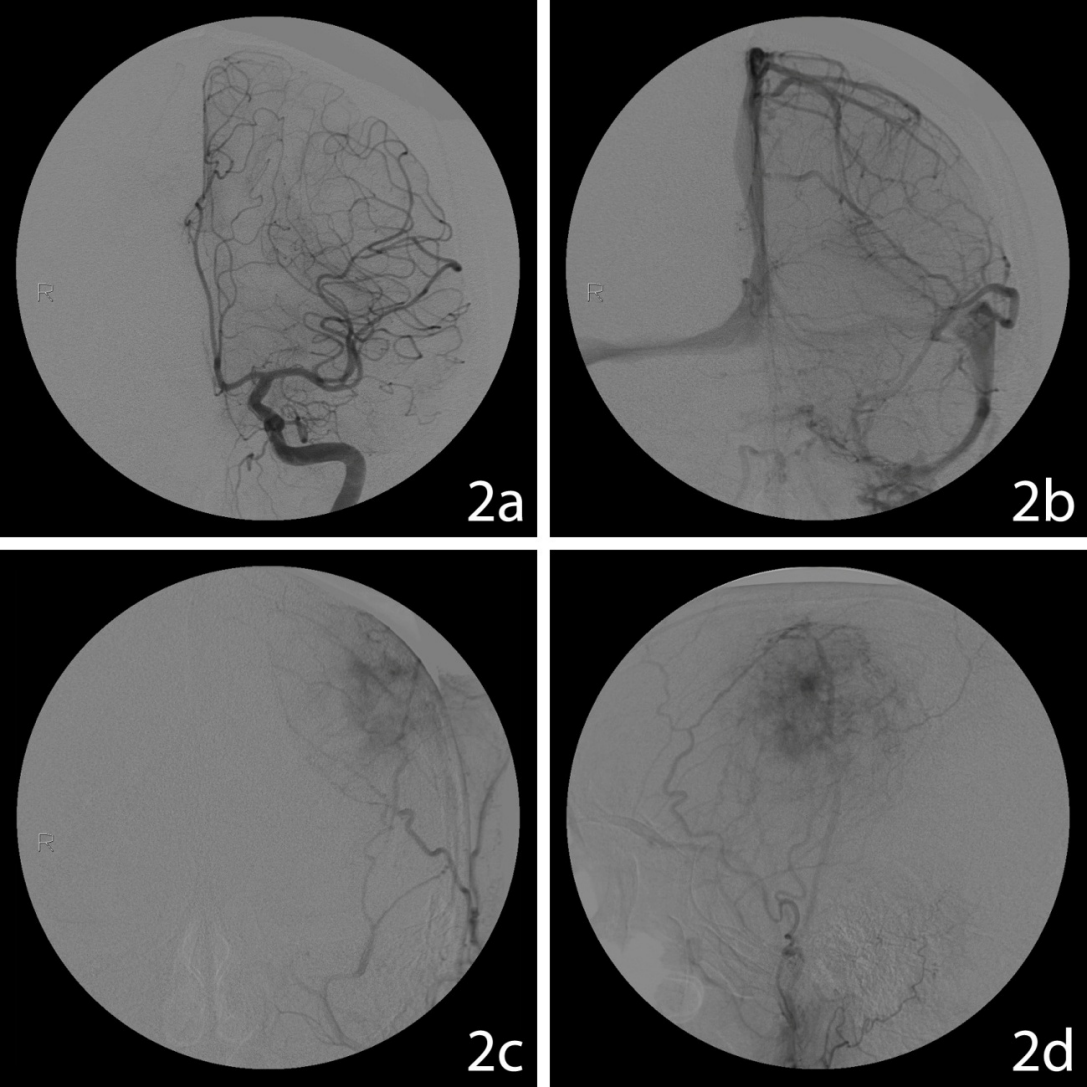
Angiography Angiography (arteriogram / venogram) from aggressive osteoblastoma. 2a: Coronal angiogram showing the branches of the left carotid artery. 2b: Coronal venogram showing the cortical veins and venous sinuses. 2c: Coronal arteriogram showing meningeal vessels. 2d: Sagital arteriogram showing meningeal vessels. The angiography evidenced a well vascularized lesion with shared blood supply from the middle meningeal artery and the superficial temporal artery. No abnormal arteriovenous shunt and no contribution from cortical arteries were observed.

We proceeded with a left temporoparietal craniotomy with a complete resection of the tumor and with grossly clean margins. The dura seemed intimately attached to both the intracranial portion of the mass and to the underlying cortex through numerous large vessels and, thus, after cauterization of the vessels, it had to be resected and replaced by a Dura-Guard dura repair patch (Baxter International, Deerfield, Illinois, US). Macroscopically, the mass contained large hemorrhagic areas, calcified nodules, and bony spicules. The rest of the tumor included yellowish connective tissue on the outer aspect and adipose-like grey tissue (Figure 3). Microscopically, the lesion was highly cellular with sheets of epithelioid osteoblasts. The final diagnosis was aggressive osteoblastoma with an extensive secondary aneurysmal bone cyst.

**Figure 4:**
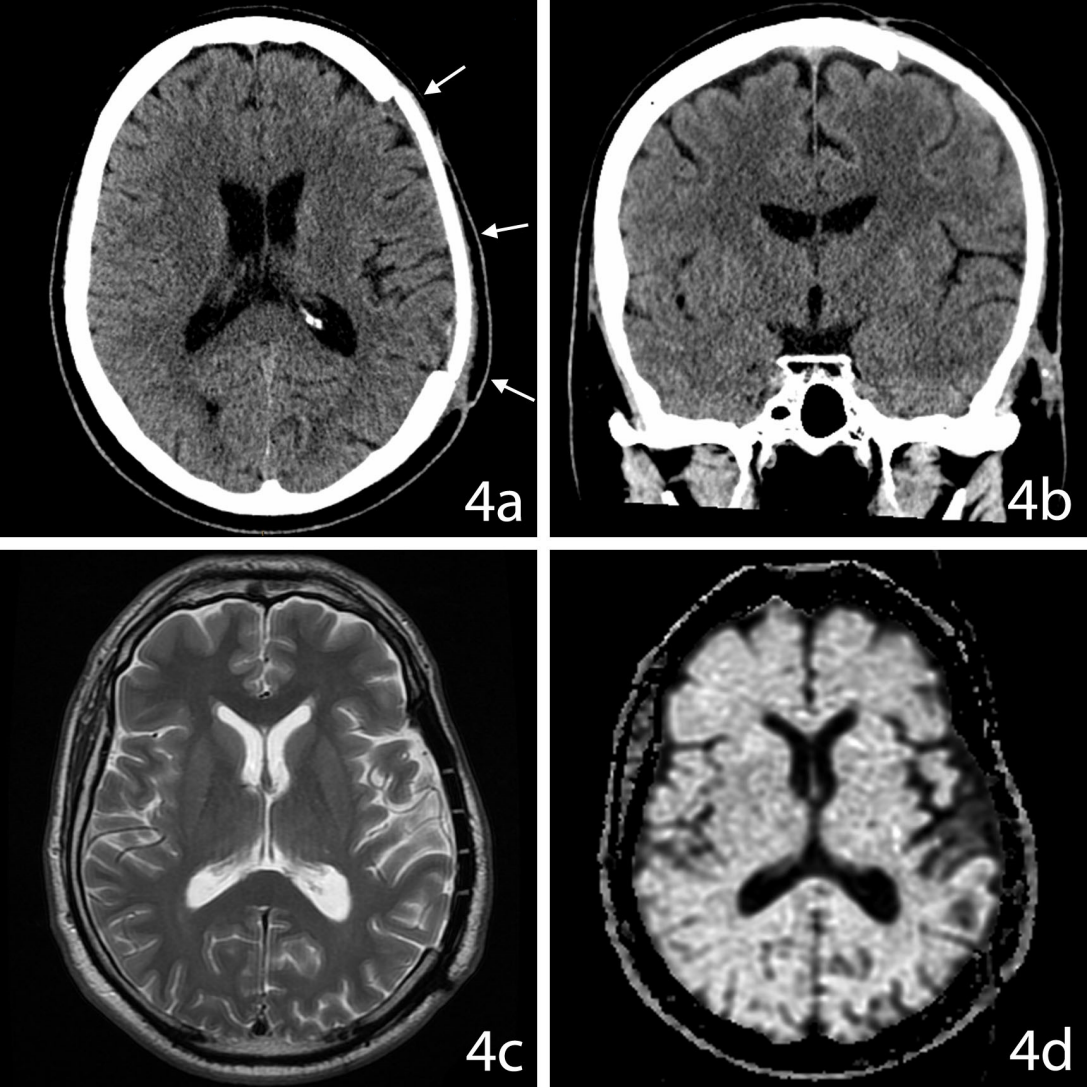
15-Month Follow-Up Imaging Aggressive osteoblastoma imaging after surgical resection and at 15 months follow-up. 4a: Axial CT. 4b: Coronal CT. 4c: Axial T2-weighted MR. 4d: Axial T1-weighted MR. The synthetic bone flap is evidenced by the white arrows (4a) on CT, which didn’t show signs of recurrent lesion (4b). A diffuse hyperintense signal is observed within the periventricular white matter on T2-weighted MR (4c). The signal changes are not from a discrete lesion, but rather vague areas of increased signal. T1-weighted MR (4d) demonstrates low intensity in the left parietal region when compared to adjacent normal cortex, consistent with encephalomalacia. There is no change in ventricle size to suggest that the signal change is related to either gliosis or mass lesion.

There was no neurological deficits postoperatively. A custom-made synthetic bone flap was inserted 56 days after the initial surgery. Follow-up at 15 months showed a stable clinical picture and no sign of local recurrence of the lesion on MRI (Figure 4).

## Discussion

Osteoblastoma is a rare locally aggressive cancer originating in the medulla of the bone with a high rate of local recurrence. The involvement of the calvarium happens only in four percent of osteoblastoma cases [2]. After reviewing the literature, we are reporting the first case of dural invasion by an aggressive osteoblatoma of the calvarium and the second one associated with aneurysmal bone cyst.

As a consequence of the rare nature of this lesion, there are no standardized management options for an aggressive osteoblastoma of the calvarium. Generally, gross total excision is adopted as the surgical treatment for aggressive osteoblastomas and could be achieved by curettage or by en bloc resection. Complete excision decreases the recurrence rate and the propensity for malignant transformation. However, in some contexts, total excision cannot be achieved due to the tumor‘s location, and adjuvant therapy may have a useful role.

## Conclusions

Aggressive osteoblastoma is a rare locally destructive bone lytic tumor, which is not included in the same spectrum of osteosarcosis. Commonly, the diagnosis cannot be made intraoperatively through frozen sections; thus, a precise histological evaluation is essential for accurate diagnosis and management. The radiological findings are variable and can be nonspecific, showing similar patterns to other tumors. We add a ninth case of aggressive osteoblastoma of the calvaria to the eight cases already reported in the literature. This is the first reported case of dural invasion.

There are no standard management options after surgical resection. Wide margin resection and duraplasty should be adopted depending on dural involvement. Adjuvant therapy is reserved for non-satisfactory resections and tumors with malignant transformation to osteosarcoma. Follow-up should be continued for at least for five years due to the risk of local recurrence.  Our patient's five-year follow-up study revealed no evidence of tumor recurrence.
